# Serum albumin to globulin ratio prior to treatment as a potential non-invasive prognostic indicator for urological cancers

**DOI:** 10.3389/fnut.2022.1012181

**Published:** 2022-10-26

**Authors:** Zhongyou Xia, Xueqin Fu, Xinzhu Yuan, Jinze Li, Hao Wang, Jing Sun, Ji Wu, Lingtong Tang

**Affiliations:** ^1^Department of Urology, Nanchong Central Hospital, The Second Clinical College, North Sichuan Medical University, Nanchong, Sichuan, China; ^2^Department of Breast Surgery, Guizhou Provincial People’s Hospital, Guiyang, Guizhou, China; ^3^Department of Nephrology, Blood Purification Center, Nanchong Central Hospital, The Second Clinical College, North Sichuan Medical University, Nanchong, Sichuan, China; ^4^Department of Urology, Institute of Urology, West China Hospital, Sichuan University, Chengdu, Sichuan, China; ^5^Department of Clinical Laboratory, The People’s Hospital of Gao County, Yibin, Sichuan, China

**Keywords:** urological cancers, albumin to globulin ratio, meta-analysis, prognosis, survival

## Abstract

**Background:**

Numerous clinical studies have reported an association between the pretreatment albumin to globulin ratio (AGR) and survival outcomes of urological cancers. However, these conclusions remain controversial. Therefore, we performed a meta-analysis to explore the prognostic value of the AGR in urinary system tumors.

**Methods:**

We retrieved eligible studies published up to June 2022 through a comprehensive search of multiple databases. Pooled hazard ratios (HRs) with 95% confidence intervals (CI) for overall survival (OS), cancer-specific survival (CSS), recurrence-free survival (RFS), progression-free survival (PFS), and biochemical recurrence-free survival (BRFS) were used to evaluated the predictive effect of the AGR before treatment in urinary system tumors. Heterogeneity test, random-effects models, fixed-effects models and sensitivity tests were used for analyses.

**Results:**

A total of 21 studies with 18,269 patients were enrolled in our meta-analysis. We found that patients with urinary system cancer with low AGR prior to treatment had poor OS [HR = 1.93, 95% CI (1.56–2.39), *p* < 0.001], CSS [HR = 2.22, 95% CI (1.67–2.96), *p* < 0.001], RFS [HR = 1.69, 95% CI (1.29–2.22), *p* < 0.001], and PFS [HR = 1.29, 95% CI (0.54–3.07), *p* < 0.001]. For prostate cancer (PCa), a low pretreatment AGR was associated with poor BRFS [HR = 1.46, 95% CI (1.28–1.67), *p* < 0.001]. Also, a subgroup analysis, stratified by ethnicity, cancer type, cutoff value, sample size and publication year, was conducted. The results showed that worse OS and CSS were significantly associated with these factors.

**Conclusion:**

Our meta-analysis revealed that the AGR before treatment could be used as a non-invasive predictive biomarker to evaluate the prognosis of urological cancer patients in clinical practice.

## Key messages

- Many studies have reported the association between the pretreatment albumin to globulin ratio (AGR) and prognosis of urological cancers, and these conclusions remain controversial.
- Meta-analysis was conducted for evaluating the prognostic value of pretreatment AGR for patients with urological cancer.
- AGR prior to treatment can be used to predict the prognosis of patients with urological cancer.

## Introduction

According to cancer statistics, in 2022 approximately 1,918,030 cancer cases will be diagnosed worldwide; cancer is still one of the leading causes of death (1). Urinary system cancers (prostate cancer, renal cancer, bladder cancer and upper tract urothelial cancer), belonging to the ten leading cancer types of diagnosed malignances; the incidence and death rates of these four urological carcinomas are increasing each year, especially in both developing and developed countries (2, 3). Over the past decades, despite considerable advances in early detection and surgical techniques, and medical therapies (e.g., chemotherapy, radiotherapy, targeted therapy, and immunotherapy) used in urinary system cancers, the 5-year survival outcome of patients diagnosed with urinary tumors remains poor; and the risk of recurrence and progression of these tumors is high (4). Numerous clinical studies have evaluated the prognosis of patients with urological cancers and have assisted clinicians in making follow-up treatment protocols using TNM stage, grade, tumor size, symptoms, and paraneoplastic syndromes (5–7). However, using the above clinical prognostic indicators alone can not accurately assess the extent of disease extent or define prognosis (7, 8). Hence, reliable, non-invasive and cost-effective pretreatment prognostic biomarkers need to be identified to evaluate the prognosis of urinary cancers and guide clinical individualized clinical treatment.

In recent years, accumulating evidence has shown that several preoperative blood-based biomarkers, such as the De Ritis ratio, neutrophil-lymphocyte ratio (NLR), serum albumin, platelet–lymphocyte ratio (PLR), and lymphocyte–monocyte ratio (LMR) have been verified as an independent prognostic indicators for patients with urinary system tumors (9–12). Recent epidemiological studies have indicated that cancer-related inflammation and nutritional status are closely associated with tumorigenesis, tumor progression, and oncological outcomes (13). The major components of serum proteins, serum albumin and globulin are valuable predictors in cancer and play a crucial role in inflammation and immunity (14, 15). The albumin-to-globulin ratio (AGR) is calculated as the albumin level divided by the globulins level. Previous studies have reported that pretreatment AGR is inversely associated with poor survival in patients with genitourinary malignant tumors (16–20). However, according to the published articles, the use of pretreatment AGR as a prognostic biomarker remains controversial. Pradere et al. (16) failed to demonstrate that upper tract urothelial carcinoma (UTUC) patients with low pretreatment AGR were associated with overall survival (OS) and recurrence-free survival (RFS). Therefore, in this context, our meta-analysis aimed to use the related published studies to explore the prognostic value of pretreatment AGR in urological cancers, to make individualized clinical decisions, and to improve patients survival.

## Materials and methods

### Literature search and eligibility criteria

Eligible citations were retrieved from Web of Science, PubMed, Google Scholar, and Cochrane Library up to June of 2022. Additionally, we also conducted a manual search of the references of the relevant studies. All searches were limited to human studies and no language restrictions were applied.

On the basis of our research objectives, the following search terms were used: (“albumin to globulin ratio” OR “albumin/globulin ratio” OR “AGR”) and (“urinary malignancy” OR “urinary neoplasm” OR “urinary cancer” OR “urinary tumor” OR “renal malignancy” OR “renal neoplasm” OR “renal cancer” OR “renal tumor” OR “bladder malignancy” OR “bladder neoplasm” OR “bladder cancer” OR “bladder tumor” OR “upper tract urothelial malignancy” OR “upper tract urothelial neoplasm” OR “upper tract urothelial cancer” OR “upper tract urothelial tumor” OR “prostate malignancy” OR “prostate neoplasm” OR “prostate cancer” OR “prostate tumor” OR “testicular malignancy” OR “testicular neoplasm” OR “testicular cancer” OR “testicular tumor” OR “transitional cell malignancy”). Since we included published articles, there was no need for review by an ethics committee. Our meta-analysis was conducted according to the PRISMA (Preferred Reporting Items for Systematic Reviews and Meta-Analyses) guideline (21).

Studies had to meet the following inclusion criteria: (1) patients were confirmed as urinary cancer by histopathology; (2) studies reported the correlation between pretreatment AGR and prognosis of patients with urinary cancer; (3) all the studies provided hazard ratios (HR) and related 95% confidence intervals (CIs) of survival outcomes, including overall survival (OS), cancer-specific survival (CSS), recurrence-free survival (RFS), progression-free survival (PFS), or biochemical recurrence-free survival (BRFS); and (4) studies with randomized controlled trials (RCTs), case-control studies, or cohort studies. The exclusion criteria were as follows: (1) duplicated studies; (2) conference abstracts, reviews, letters and case reports; (3) basic experiments and animal researches; and (4) studies with unavailable data.

### Quality evaluation

In this study, the Newcastle-Ottawa Scale (NOS), which includes three domains (selection of the cohort, comparability of the groups, and quality of the outcomes), was used to assess the quality of the included studies (22). The NOS scale has nine stars, and a score of six or more was considered a high-quality study in our meta-analysis.

### Data extraction

The primary basic information for each included study was as follows: first author’s name, country, study design, sample size, intervention, age, cancer type, cut-off value for AGR, analysis method, and follow-up time. In addition, the pooled HRs and corresponding 95% CIs of the survival outcomes (OS, CSS, RFS, PFS, and BRFS) were extracted. As a common index to evaluate the prognosis of cancer patients, OS is only concerned about whether the patient dies, not the specific cause of death and the follow-up time is long. The other tumor-specific prognostic indexes, such as CSS, RFS, PFS, or/and BRFS, are supplement to OS for better manage tumor patients. Therefore, in order to better evaluate the clinical efficacy of AGR in the prognosis of urinary cancers, we made a comprehensive analysis of these indexes. If univariate and multivariate analyses were conducted in a study, we extracted the multivariate analysis data for follow-up analysis.

The above steps independently performed by two authors, and a third author resolved any discrepancies.

### Statistical analysis

All the statistical data were processed by Stata 16 (StataCorp LP, College station, TX, United States of America LP, University City, TX, USA). For studies that provided only the Kaplan-Meier curves, we used Engauge Digitizer 4.1 software to extract the relevant survival data. The HRs and corresponding 95% CIs of the included articles were extracted to assess the prognostic significance of the AGR in urological cancers. Cochrane’s *Q* test and Higgin’s I^2^ test were used to measure the heterogeneity among the included studies. According to the results of the heterogeneity test, the random-effects model was utilized with high heterogeneity (I^2^ ≥ 50% or *p* < 0.1). Otherwise, fixed-effects models were used for the analyses. Sensitivity analysis, involving the removal of each individual study, was also used to assess the reliability and stability of our survival outcomes. Additionally, Begg’s test was performed to identify potential publication bias across studies if ten or more articles were included in meta-analysis. A value of *P* less than 0.05 was considered as a statistical significance.

## Results

### Search results and study characteristics

**FIGURE 1:**
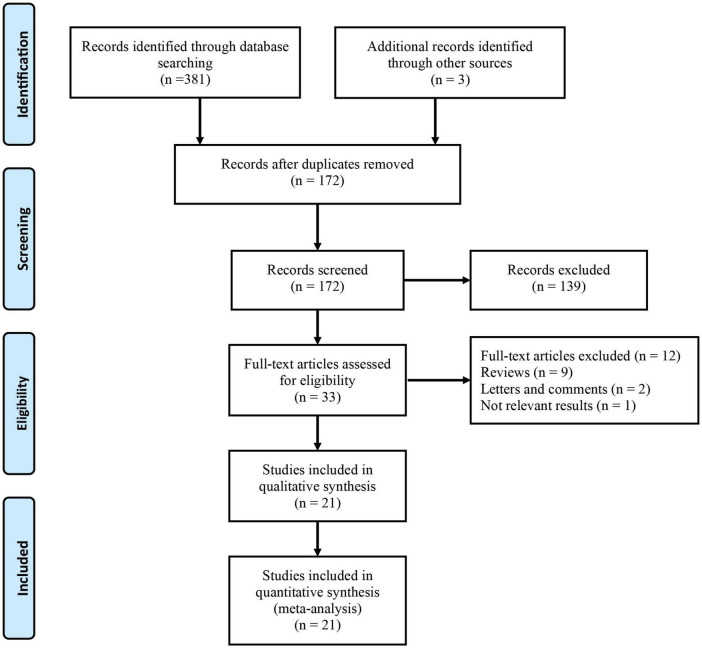
Flow diagram of the study selection process.

Figure 1 shows the detailed screening process used in this meta-analysis. Based on the search of electronic databases, 384 published articles were initially found, and 172 studies remained after removing 212 duplicates. After screening the titles and abstracts, 33 studies remained for further evaluation, and their full texts were read for eligibility. Twelve studies were excluded from the full-text analysis for the following reasons: nine reviews, two letters and comments, and one did not provide available data. Finally, there were 18,269 patients who were included in the 21 studies (16–19, 23–39)in this meta-analysis.

From 2017 to 2022, all the eligible studies were published, with a sample size between 104 and 6,041 participants and the cutoff values of AGR ranged from 1.11-1.6. Notably, all the included studies were only case-control, six of the 21 studies were conducted in a single center, and 15 studies were conducted in multiple centers. Of the included studies, five studies reported the correlation between pretreatment AGR and prognosis of bladder cancer (BC), seven articles explored upper tract urothelial carcinoma (UTUC), five investigated renal cell cancer (RCC), and two focused on prostate cancer (PC). Using the NOS score, eighteen articles scored 7-9 on the NOS and three studies scored 5 or 6 on the NOS, indicating high quality of the included articles. Table 1 summarizes the characteristics of the included articles.

**TABLE 1:** Baseline characteristics of include studies and methodological assessment.

Authors (year)	Country	Study design	Sample size	Intervention	Age^a^	Cancer type	Stage	Cutoff	Follow-up time^c^ (months)	Endpoint	Quality score
Zhang et al. (29)	China	Retrospective	187	RNU	70 (61–74)	UTUC	N	1.45	Median 78 (32-92)	OS, CSS	8
Liu et al. (36)	China	Retrospective	296	RC	61.71 ± 11.08	BC	N	1.6	Median 72.0 (49.75-115.50)	RFS, CSS	7
Liu et al. (37)	China	PSM	104	RC	NA	BC	N	1.55	Median 38 (1–90)	OS, PFS, TSS	7
Chen et al. (24)	China	PSM	592	RN and PN	NA	RCC	N	1.22	Median 42.3 (3–50)	OS, CSS	7
He et al. (18)	China	Retrospective	895	RN or PN	51.44 ± 13.44	RCC	N	1.47	Median 69.68 (95%CI: 65.73–73.63)	OS	7
Fukushima et al. (30)	Japan	Retrospective	105	RNU	74 (49–89)	UTUC	N	1.24	Median 46 (22–83)	OS, DFS	6
Otsuka et al. (31)	Japan	Retrospective	124	RNU	69 (64–75)	UTUC	N	1.4	Median 55 (28–)	OS, RFS, CSS	7
Koparal et al. (25)	Turkey	Retrospective	162	RN and PN	56.5 ± 11.8	RCC	N	1.4	Median 27.5 (6–89)	OS, DFS	6
Xu et al. (32)	China	Retrospective	620	RNU	NA	UTUC	N	1.45	Median 50 (28–78)	RFS, CSS, OS	8
Niwa et al. (19)	Japan	Retrospective	364	TUR	71 (63–77)	BC	N	1.6	Median 47 (18–89)	RFS, PFS	7
Omura et al. (33)	Japan	Retrospective	179	RNU	75 (66–79)	UTUC	N	1.25	Median 34 (17–63)	OS, CSS	7
Chung et al. (26)	Korea	Retrospective	2970	RN or RPN	55.6 ± 13.2	RCC	N	1.47	Median 26.0 (9.0–59.0)	OS, RFS	8
Oh et al. (38)	Korea	Retrospective	176	RC	68.05 ± 8.96	BC	N	1.32	Median 32.4 (0.2–95.3)	CSS, MFS	8
Quhal et al. (39)	multicenter	Retrospective	1096	TURBT	67 (58–74)	BC	N	1.41	Median 63.7 (25.3–111)	PFS, RFS	8
Miura et al. (34)	multicenter	Retrospective	2492	RNU	69 (27–97)	UTUC	N	1.4	Median 38	RFS, CSS, OS	7
Pradere et al. (16)	multicenter	Retrospective	172	NAC + RNU	68 (63–73)	UTUC	N	1.42	Median 26 (11–56)	OS, RFS	8
Taguchi et al. (35)	multicenter	Retrospective	176	pembrolizumab	71 (66–76)	Mix^b^	N + M	0.95	Median 7.5 (4–14)	OS, CSS, PFS	7
Laukhtina et al. (28)	multicenter	Retrospective	613	RN	57 (50–64)	mRCC	M	1.43	Median 31 (16–58)	OS, CSS	8
Aktepe et al. (27)	Turkey	Retrospective	163	Target therapy	60 (53–65)	mRCC	M	1.11	NA	OS, PFS	6
Aydh et al. (23)	multicenter	Retrospective	6041	RP	61(57–66)	PCa	N	1.31	Median 45 (35–58)	BRFS	8
Chung et al. (17)	Korea	Retrospective	742	RP	NA	PCa	N	1.53	NA	BRFS	8

^a^Age, Mean ± SD/Mean (Range).

^b^Mix, bladder cancer, upper tract urothelial carcinoma.

^c^Follow-up Time, median (range)/median; PSM, propensity score-matched; RNU, radical nephroureterectomy; RN, radical nephrectomy; PN, partial nephrectomy; BC, bladder Cancer; UTUC, upper tract urothelial carcinoma; RCC, renal cell carcinoma; PCa, prostate cancer; N, non-metastatic; N + M, non-metastatic + metastatic; TURBT, Transurethral resection of bladder tumor; NAC, neoadjuvant chemotherapy; OS, overall survival; CSS, cancer-specific survival; RFS, recurrence-free survival; TSS, tumor-specific survival; PFS, progression-free survival; MFS, metastasis-free survival; NA, age data was not available.

### Prognostic significance of the albumin to globulin ratio for overall survival

**FIGURE 2:**
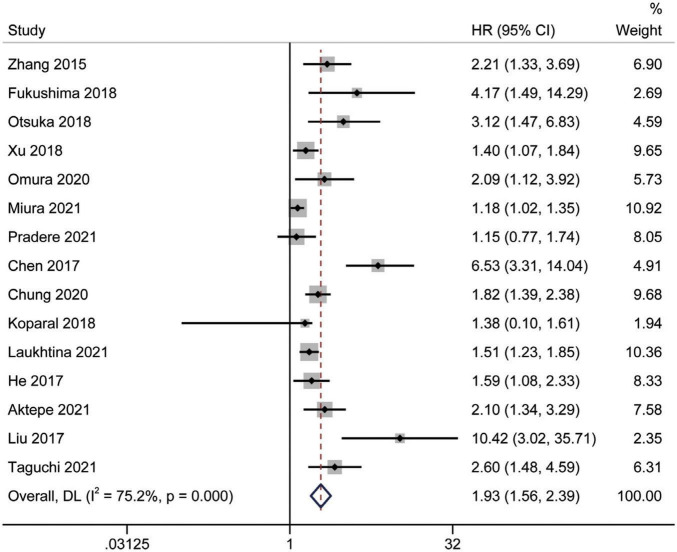
Forest plot reflects the association between AGR and OS for urological cancers.

**FIGURE 3:**
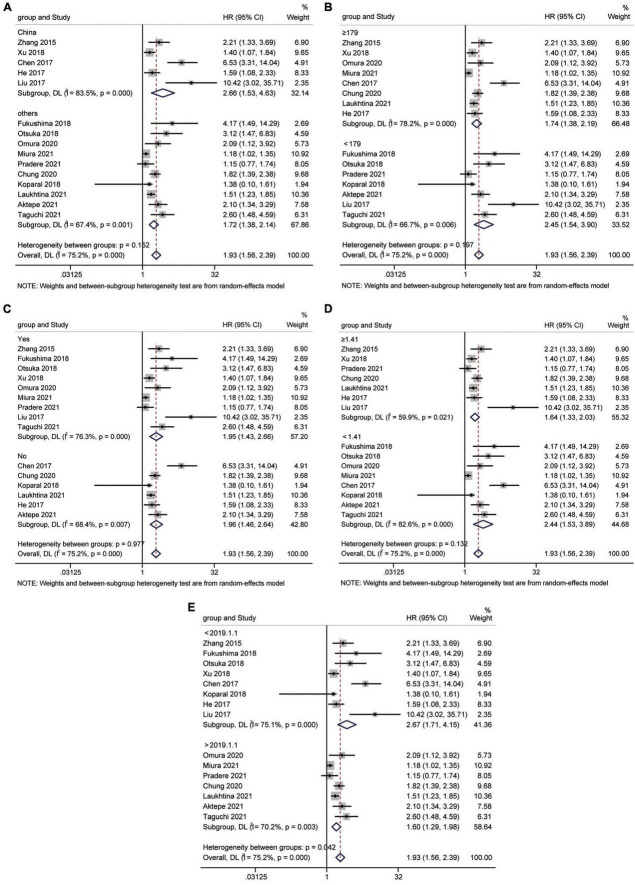
Forest plot for subgroup analysis of OS and AGR. **(A)** Subgroup analysis of OS and AGR according to ethnicity; **(B)** Subgroup analysis of OS and AGR according to sample size; **(C)** Subgroup analysis of OS and AGR according to urothelial carcinoma (yes or no); **(D)** Subgroup analysis of OS and AGR according to cut-off values; **(E)** Subgroup analysis of OS and AGR according to publication year.

A total of 15 studies (16, 18, 24–35, 37) involving 9,554 patients revealed an association between AGR and OS in a multivariate analysis of urological cancers. According to Figure 2, patients with low pretreatment AGR had a worse survival outcomes than those with high AGR [HR = 1.93, 95% CI (1.56–2.39), *p* < 0.001]. Because of the high heterogeneity between the studies (I^2^ = 75.2%, *p* < 0.001), we used a random-effects model. To clarify the source of heterogeneity between studies, we performed subgroups analysis based on ethnicity, sample size, urothelial carcinoma (yes or no), AGR cut-off values and publication year. The results of the subgroups analysis also supported the fact that low AGR was related to poor OS and revealed that ethnicity, sample size, urothelial carcinoma (yes or no), AGR cut-off values, and publication year might be causes of high heterogeneity (Figure 3).

### Prognostic significance of the albumin to globulin ratio for cancer-specific survival

**FIGURE 4:**
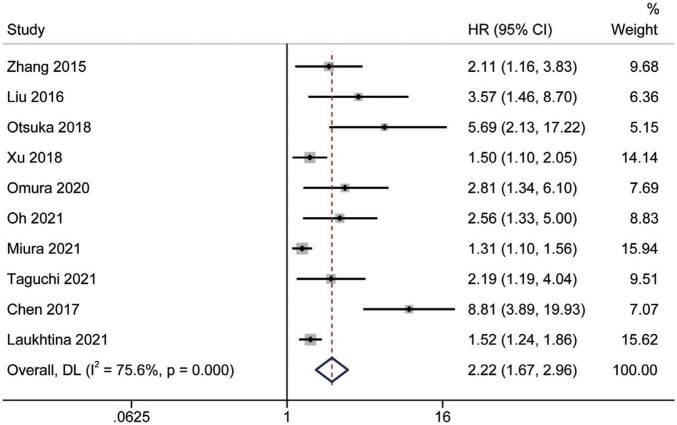
Forest plot reflects the association between AGR and CSS for urological cancers.

**FIGURE 5:**
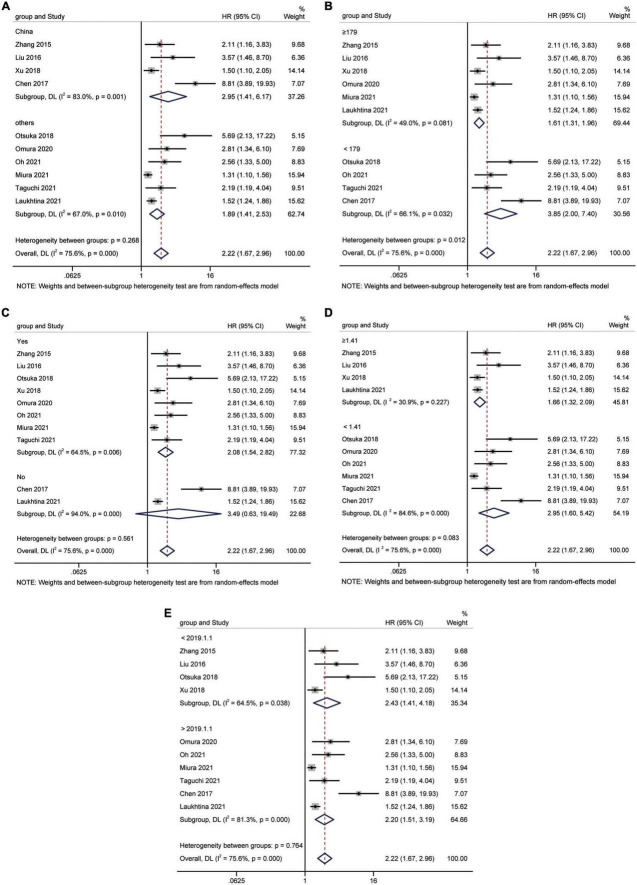
Forest plot for subgroup analysis of CSS and AGR. **(A)** Subgroup analysis of CSS and AGR according to ethnicity; **(B)** Subgroup analysis of CSS and AGR according to sample size; **(C)** Subgroup analysis of CSS and AGR according to urothelial carcinoma (yes or no); **(D)** Subgroup analysis of CSS and AGR according to cut-off values; **(E)** Subgroup analysis of CSS and AGR according to publication year.

Study reports from 10 studies (24, 28, 29, 31–36, 38), with 5,455 patients enrolled, indicated the prognostic value of AGR before treatment in patients with urinary system cancers on CSS. Because of Cochrane’s *Q* test and *I*^2^ test revealed substantial heterogeneity among studies (*I*^2^ = 75.6%, *p* < 0.001), a random-effects model was used to combine the HR of each study. Pooled analysis showed that decreased pretreatment AGR was significantly associated with shorter CSS [HR = 2.22, 95% CI (1.67–2.96), *p* < 0.001] (Figure 4). Subgroups analysis stratified by ethnicity, sample size, urothelial carcinoma (yes or no), AGR cut-off values and publication year, and results showed that low pretreatment AGR was associated with poor CSS (Figure 5).

### Prognostic significance of the albumin to globulin ratio for recurrence-free survival

**FIGURE 6:**
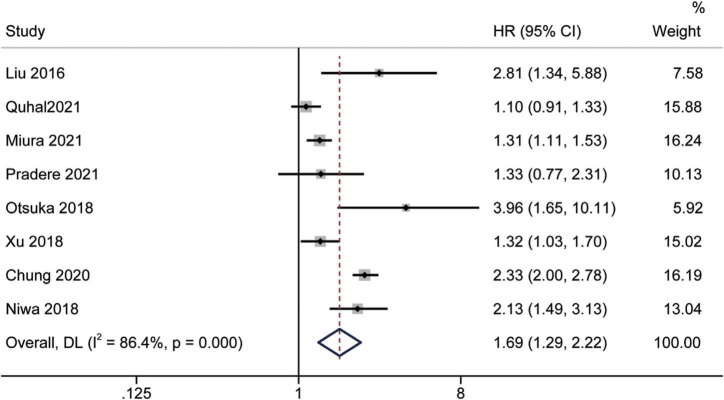
Forest plot reflects the association between AGR and RFS for urological cancers.

Eight studies (16, 19, 26, 31, 32, 34, 36, 39) assessed the prognostic impact of AGR before treatment on RFS using multivariate analysis. Since the statistics showed considerable inter-study heterogeneity (I^2^ = 86.4%, p < 0.001), a random-effects model was used. The pooled meta-analysis results showed that low pretreatment AGR could predict inferior RFS independently [HR = 1.69, 95% CI (1.29–2.22), *p* < 0.001] (Figure 6).

### Prognostic significance of the albumin to globulin ratio for progression-free survival and biochemical recurrence-free survival

**FIGURE 7:**
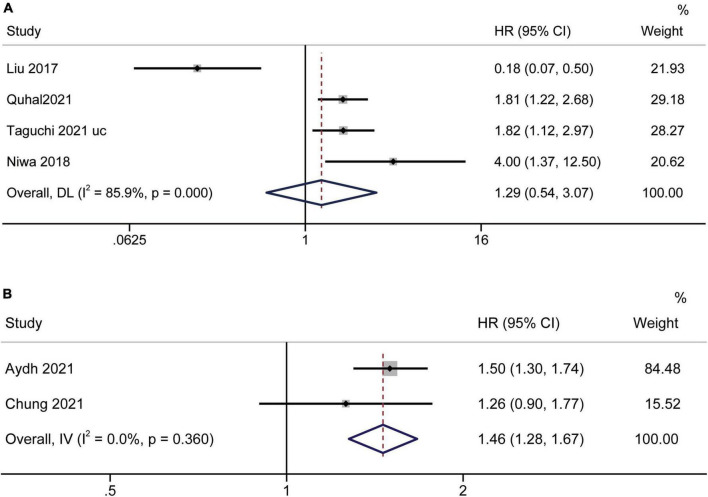
Forest plot reflects the association between AGR and PFS/BRFS for urological cancers. **(A)** AGR and PFS; **(B)** AGR and BRFS.

Limited related data from four studies (19, 35, 37, 39) and two studies (17, 23) were suitable for PFS and BRFS analyses, respectively. Analysis of datasets revealed an association between low AGR and worse PFS [HR = 1.29, 95% CI (0.54–3.07), *p* < 0.001] (Figure 7A), and poor BRFS [HR = 1.46, 95% CI (1.28–1.67), *p* < 0.001] (Figure 7B).

### Sensitivity analysis

**FIGURE 8:**
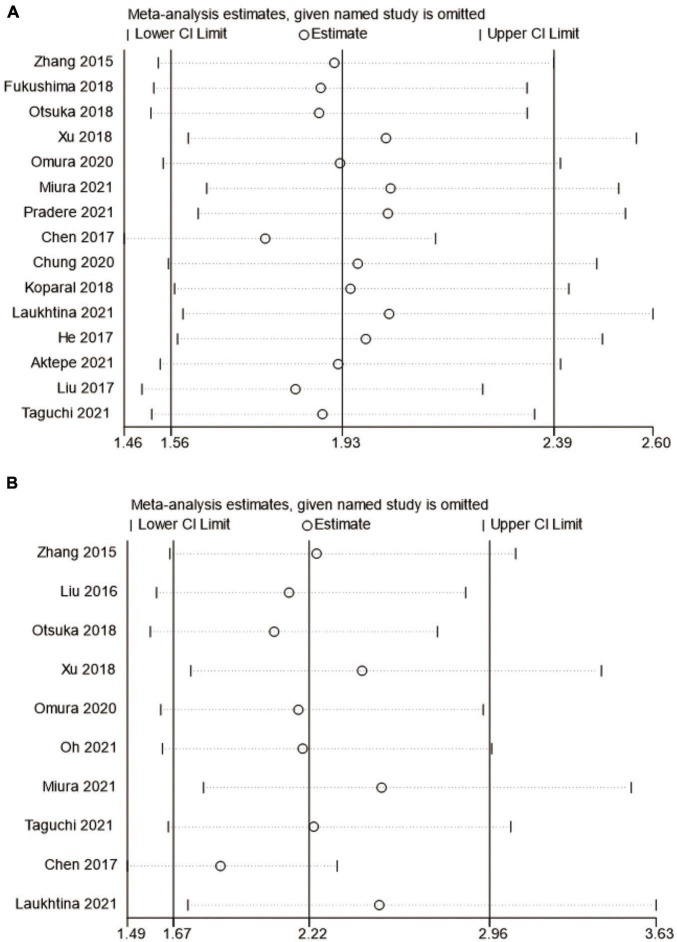
Forest plot for sensitivity analysis. **(A)** overall survival and **(B)** cancer-specific survival.

In this meta-analysis, we conducted a sensitivity analysis of OS and CSS outcomes to ascertain the strength of our results. The pooled HRs of OS and CSS were not significantly affected when the study was removed. Therefore, we believe that our results are reliable (Figure 8**).**

### Publication bias

**FIGURE 9:**
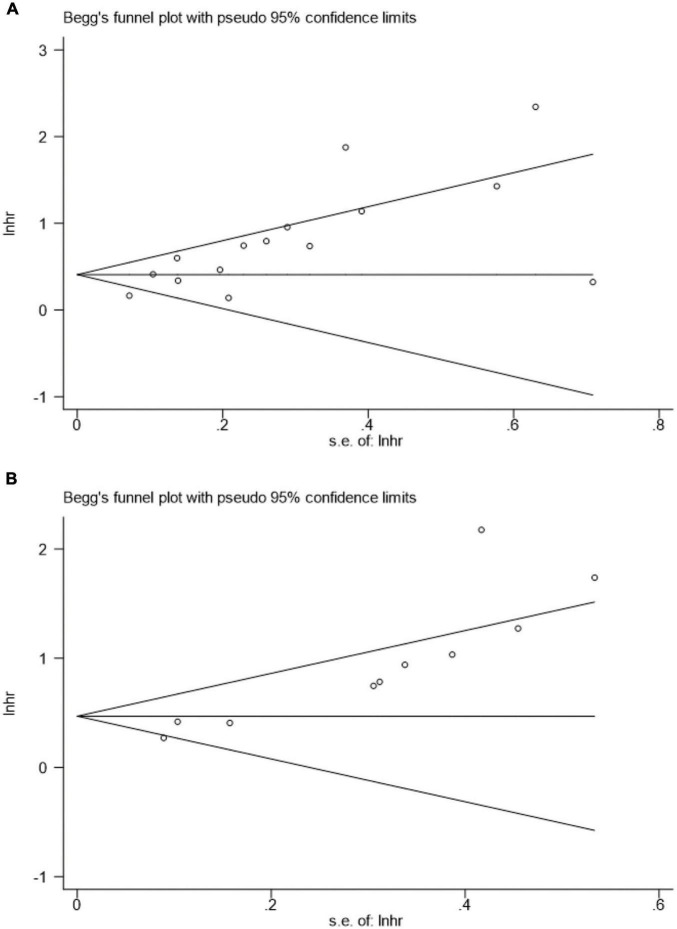
Begg’s test for **(A)** overall survival and **(B)** cancer-specific survival.

Figure 9 presents the funnel plots for OS and CSS, and asymmetry was observed by visual inspection of the Begg’s funnel plots. Therefore, a potential publication bias may have was existed for the association of pretreatment AGR, OS, and CSS based on funnel plots. Our Begg’s statistical tests aslo indicated that there was a significant publication bias (OS, *p* = 0.018; CSS, *p* < 0.001).

## Discussion

Recurrence and metastasis of urinary tumors are common and seriously affect both the prognosis and quality of life of patients. Approximately 75% of high-grade non- muscle-invasive bladder cancer (NMIBC) cases would relapsed, progressed or died within 5 years (40). For urothelial cancer, even if the patients undergo radical surgery, the 5-year survival rate is less than 50% (41). Furthermore, 20% to 30% of patients with RCC undergoing curative resection have distant metastasis during follow-up, and the 5-year survival rate is very low (5-10%) (42). Therefore, it is of great significance to identify the predictive indicators that affect the prognosis and treatment decision-making for urinary cancer. To our knowledge, this is the first and most comprehensive meta-analysis to evaluate the prognostic impact of AGR on UC.

As a promising blood marker, some studies have reported a relationship between pretreatment AGR and urological cancers up to now. Owing to the inconsistent results of these studies, we iperformed a meta-analysis of twenty-one published studies involving 18,269 patients to further evaluate the potential value of AGR for predicting the survival of patients with urinary cancer. Based on the pooled data, our results are in line with the most of the related published literatures, which demonstrated that low pretreatment AGR is an independent predictor of survival outcomes. With a decrease in AGR, patients with urinary system cancer (BC, UTUC, RCC, and PCa) had worse OS, CSS, RFS, PFS, and BRFS outcomes. We then performed a subgroups analysis of OS and CSS based on ethnicity, cancer type, cutoff value, sample size or publication year. The results of the subgroup analysis were consistent with meta-regression analyses. Finally, sensitivity analyses also demonstrated the robustness of our outcomes. Albumin and globulins are the two most abundant proteins in human blood plasma, and can be easily and cost-effectively measured. Thus, AGR can be used as a competent prognostic biomarker in patients with urological cancer.

Growing evidence suggests that inflammation in the tumor microenvironment plays a critical role in tumor growth, progression and metastasis (43). Tumor growth, necrosis and hypoxia trigger the production of a series of inflammatory factors, such as tumor necrosis factor (TNF), interleukin-1 and interleukin-6, which increase vascular permeability by damaging vascular endothelial cells (44). In 2014, Duran and his colleagues first demonstrated that the AGR was a strong prognostic indicator of poor survival outcomes in lung adenocarcinoma patients (45). Although the specific mechanism is not clear, it is at least related to these two indicators. Albumin is the main component of total serum protein, and its level either reflects the body’s nutritional status or represents the systemic inflammation (18). Cytokines and chemokines produced by tumor cells can suppress albumin production and lead to malnutrition, which accelerates disease progression (46). Moreover, studies have reported that albumin plays an important role in delivering chemotherapy drugs to cancer patients, and this mechanism affects the survival outcomes (47). Furthermore, globulins, including complementary components, C-reactive protein (CRP), and immunoglobulin, is also involved in the inflammatory responses and immunosuppression against cancer cells in the human body (48). Chronic inflammation can affect not only the tumor growth but also angiogenesis and cancer migration (49). Studies have shown that some inflammatory markers, CRP and NLR, were closely related to the prognosis of cancer patients (50, 51). Equally, the AGR is also a inflammatory biomarker and decrease of AGR reflects the malnutrition and inflammatory response. Because of the AGR incorporates the advantages of albumin and globulin, and it is more likely to predict the poor survival in cancer patients.

Previous meta-analyses have explored the correlation between AGR and different organs and systems. A meta-analysis conducted by Li et al. (52) discovered that a decreased AGR suffer from worse OS and DFS in patients with lung cancer. In colorectal cancer, Ma et al. (53) provided evidence that low pretreatment AGR was related to poor OS (HR = 2.07, *P* < 0.01) and DFS/PFS (HR = 2.10, *P* = 0.01), and advanced clinicopathological features, including age, tumor size, node metastasis stage, and tumor depth. Additionally, many recent studies have explored the association between the AGR and genitourinary cancers. Omura and his colleagues performed a retrospective study involving 179 patients with UTUC who underwent radical nephroureterectomy and revealed that a low AGR could predict the prognosis of patients with non-metastatic UTUC (33). A multicenter research team found that the UTUC patients with a low pretreatment AGR had a markedly shorter OS and RFS than those with a high AGR patients (16). In Korea, Chung’s study proved that the association between the preoperative serum AGR and the poor prognosis in patients with RCC in a large cohort (26). For non-metastatic PCa patients who received radical prostatectomy (RP), the findings of Aydh et al. and Chung et al. indicated that the pretreatment AGR can be a useful serological marker for predicting the BRFS and adverse pathology (17, 23). In 2021, Zhang et al. (48) verified that the AGR combined with other indices [C-reactive protein/albumin ratio (CAR), neutrophil-lymphocyte ratio (NLR), and other clinicopathological features] could predict OS and PFS in BC patients after radical cystectomy. This is the first meta-analysis to assess the prognostic value of the AGR in urinary system cancers. Our results further showed that a low pretreatment AGR indicates poor prognosis, which is in line with previous studies.

Although this meta-analysis provides evidence regarding the prognostic value of AGR in patients with urological cancer, several limitations cannot be avoided. First, most of the included studies were single-center and retrospective studies; therefore, the heterogeneity between studies is inevitable. Second, because of different treatment strategies for various cancers may introduce bias in the results. Third, the cutoff threshold of AGR selected by different studies was different, making it difficult to determine the optimal cutoff value. Fourth, only common urological tumors were included in this study, but the correlation between AGR and the prognosis of other genitourinary tumors is still unclear. Finally, considering the limitation of many factors affecting the prognosis of urinary cancers, the evaluation effectiveness of this index needs to be verified. Therefore, further studies need to be conducted.

## Conclusion

In summary, our study proved that a low AGR before treatment is associated with inferior OS, CSS, RFS, PFS, and BRFS outcomes in urinary system cancers. As a non-invasive, effective, and cost-effective indicator, the AGR can be used to predict the prognosis of patients with urological cancer. However, further large-scale prospective studies with larger sample sizes are needed.

## Data availability statement

The original contributions presented in this study are included in the article/supplementary material, further inquiries can be directed to the corresponding author/s.

## Author contributions

LT and JW conceived and designed the experiments. ZX, XF, JL, and XY analyzed the data. JL, JS, and HW contributed the reagents, materials, and analysis. ZX and XF wrote the manuscript. All authors contributed to the article and approved the submitted version.
